# An animal component-free bioprocess for synthesizing 3D human matrix scaffolds using mesenchymal stromal cells

**DOI:** 10.3389/fcell.2026.1624745

**Published:** 2026-04-13

**Authors:** Shaianne N. Stein, Lorena R. Braid

**Affiliations:** 1 Simon Fraser University, Department of Molecular Biology and Biochemistry, Burnaby, BC, Canada; 2 Simon Fraser University, Centre for Cell Biology, Development and Disease, Burnaby, BC, Canada

**Keywords:** animal component-free, basement membrane, collagen, decellularized matrix, extracellular matrix, mesenchymal stromal cells (MSCs), spheroid

## Abstract

**Introduction:**

The basement membrane is a specialized extracellular matrix that compartmentalizes epithelial and endothelial tissues and provides essential structural and signaling cues for tissue organization. Whereas fibrillar collagens (Col) of the interstitial matrix (such as Col I and Col III) are widely used in tissue modeling, the networking collagens that scaffold the basement membrane, including human Col IV and Col VI, remain difficult to access. Commercial basement membrane surrogates such as Matrigel® are derived from murine tumors and are ill defined, dilute, variable, and incompatible with animal-free biomanufacturing. Thus, there is a crucial need for human-derived basement membrane matrices that are free of xenogenic contaminants and do not rely on breeding animals.

**Methods:**

Here, we analyzed whether human mesenchymal stromal cells (MSCs) could serve as a platform to produce self-assembling basement membrane components under chemically defined, xeno-free conditions. MSCs from placental, umbilical cord, bone marrow, and adipose tissues were cultured as three-dimensional spheroids and adherent multilayered sheets.

**Results:**

Confocal imaging of whole-mount, decellularized spheroid matrices showed complex networks of fibronectin (FN) and Col IV with topological and organizational features characteristic of basement membrane. Perinatal MSCs produced distinct matrix architectures consisting of apical FN sheets underlaid by continuous Col IV networks. Time-resolved imaging of umbilical cord MSC-derived matrix sheets demonstrated a reproducible sequence of basement membrane assembly that parallels developmental tissue organization.

**Discussion:**

Together, these findings demonstrate that human MSCs cultured entirely without entirely animal-derived components can synthesize functional basement membrane proteins that self-assemble into ordered, tissue-like scaffolds. In this work, we establish MSCs as a scalable, sustainable, and cruelty-free platform for manufacturing human basement membrane matrices for bioengineering and regenerative medicine applications.

## 1 Introduction

The extracellular matrix (ECM) performs a central function in tissue organization, morphogenesis, and cell fate determination, making it a critical component of bioengineered human tissues and organoid systems (Kozlowski et al., 2021; Olesen et al., 2021; Jain et al., 2022; Motta et al., 2023). Whereas interstitial matrix provides essential structural support, tensile strength, and scaffolding for cells within connective tissues, basement membrane-type matrices provide essential biochemical and structural cues that regulate epithelial and endothelial polarity, vascularization, and growth factor signaling (Yurchenco, 2011; Jayadev and Sherwood, 2017; Sekiguchi and Yamada, 2018; Khalilgharibi and Mao, 2021; Pompili et al., 2021; Jain et al., 2022). Despite their importance, the development of human-relevant basement membrane scaffolds remains limited because of the insufficient availability of appropriate matrix materials (Aisenbrey and Murphy, 2020; Perugini and Santin, 2021).

Currently, basement membrane surrogates such as Matrigel® and Cultrex™ are widely used in three-dimensional (3D) culture systems (Kane et al., 2018; Aisenbrey and Murphy, 2020). However, these materials are derived from murine Engelbreth–Holm–Swarm sarcoma and only distantly resemble the native human basement membrane in composition and organization (Kleinman and Martin, 2005; Hughes et al., 2010; Huang et al., 2021; Kozlowski et al., 2021). Their reliance on breeding animals for production raises ethical and sustainability concerns, while xenogeneic contaminants, batch-to-batch variability, and undefined bioactive components limit experimental reproducibility and translational applicability (Jung et al., 2010; Hemeda et al., 2014; Baker, 2016a; 2016b; Aisenbrey and Murphy, 2020; Liu et al., 2023). Given the harsh conditions required to solubilize tumor matrix proteins, these hydrogels likely contain fragmented ECM proteins that are incapable of physiologic matrix self-assembly (Hughes et al., 2010; Tuftee et al., 2024).

Human cell-derived ECM represents a promising alternative, as the cells can synthesize, process, and assemble matrix proteins into organized networks that more closely resemble native tissue architecture (Muiznieks and Keeley, 2013; Franco-Barraza et al., 2016; Fonseca et al., 2023; Solomonov et al., 2025). Approaches including tissue decellularization and cell-generated microtissues have demonstrated the feasibility of producing human ECM scaffolds *in vitro* (Johnson et al., 2016; Duisit et al., 2018; Chiang et al., 2021; Malakpour-Permlid et al., 2021; Jin et al., 2022; Fonseca et al., 2023; Zhang et al., 2023; Chen et al., 2025). Mesenchymal stromal cells (MSCs) are favored candidates for matrix production due to their availability from multiple tissues, robust secretory capacity, and established use in biomanufacturing (Smith et al., 2020; Tao et al., 2020; Pompili et al., 2021; Fonseca et al., 2023). However, most studies of MSC-derived ECM have relied on animal-derived or other ill-defined supplements such as fetal bovine serum (FBS) or human platelet lysate (hPL) (Amable et al., 2014; Maia et al., 2014; Novoseletskaya et al., 2020; Sagaradze et al., 2020; Yin et al., 2024; Chen et al., 2025).

The use of serum-based culture systems presents significant limitations for matrix manufacturing. Growth factors present in FBS and hPL become sequestered within the ECM, leading to the embedding of xenogeneic or donor-dependent proteins that compromise matrix definition, reproducibility, and downstream applications (Jung et al., 2010; Hemeda et al., 2014; Baker, 2016a; 2016b; Zhang et al., 2017; Keppie et al., 2021; Liu et al., 2023). Chemically defined media formulations reduce the complex growth factor milieu found in serum and could therefore be associated with reduced cellular performance (Jung et al., 2010; Hemeda et al., 2014; Xu et al., 2020; Spoerer et al., 2025). However, it must be noted that FBS-supplemented media expose cells to a fetal-like environment, which can artificially increase their growth rate, whereas the most physiologically relevant growth rate would be observed in the natural microenvironment of the body (Malakpour-Permlid et al., 2025). Recent developments offer a path toward reproducible animal component-free bioprocessing (Weber et al., 2024; Malakpour-Permlid et al., 2025; Oredsson et al., 2025), including the cultivation of 3D primary hepatocyte spheroids (Mickols et al., 2025).

Critically, matrix production requires not only the synthesis of ECM proteins but also the coordinated expression of processing enzymes necessary to convert precursor molecules, such as procollagens, into mature, self-assembling networks (Muiznieks and Keeley, 2013; Solomonov et al., 2025). It is not yet known whether chemically defined media can support this level of MSC-mediated matrix assembly.

Here, we assess whether human MSCs from multiple tissue sources can self-assemble basement membrane-type matrices under fully defined, animal component-free conditions. Using a quality-by-design approach compatible with good manufacturing practices, we show that MSCs cultured in chemically defined media form 3D spheroids and adherent multilayered sheets that deposit Col IV-positive basement membrane matrices. These matrices contain canonical basement membrane components, can be decellularized, and support recellularization, thus demonstrating their potential as scalable and human-relevant ECM scaffolds for bioengineering applications.

## 2 Materials and methods

### 2.1 Cell source

The culture age of MSCs was measured by the population doubling level (PDL), which is a more accurate metric than the passage number. As described previously, the PDL was calculated using the following formula:

$$\text{PDL}=3.32\;\left({\text{logX}}_{e}-{\text{logX}}_{b}\right)+S$$

where X_e_ is the final viable cell count, X_b_ is the initial seeded cell count, and S is the PDL at the start of the culture incubation (Wiese et al., 2019). Early-passage MSCs (passage 3–5), as used in this study, double approximately every 24 h (Wiese et al., 2019).

High-quality human MSCs were obtained from commercial sources. Umbilical cord (UC) MSCs were provided by Tissue Regeneration Therapeutics (TRT), Inc. (Toronto, ON, Canada). UC MSCs were isolated from the perivascular Wharton’s jelly of a healthy term (>37 weeks) UC from one male infant delivered by cesarean section and cryopreserved at a mean PDL (mPDL) of 4.87.

Adipose (AD) and bone marrow (BM) MSCs from female donors were obtained from RoosterBio Inc. (Frederick, MD, United States) at 8.9 and 8.8 mPDL, respectively. Placental (PL) MSCs from a female donor were obtained from OrganaBio, LLC. (Miami, FL, United States) at mPDL 8.39. Experiments were performed using one donor population per tissue of origin. For whole-mount immunostained spheroids (Section 2.7), three UC MSC donor populations were pooled and cultivated.

### 2.2 Cell culture and maintenance

For cell expansion, MSCs were seeded at a density of 1,333 cells (UC MSCs) and 3,000 cells (AD, BM, and PL MSCs)/cm^2^ in chemically defined phenol red-free MSC NutriStem XF® complete growth media (Sartorius AG, Bohemia, NY, United States). Cells were seeded according to TRT and RoosterBio’s recommended seeding density and expansion protocols that have been optimized for maximal cell expansion. Culture-ware was pretreated with 2.67 μg/cm^2^ Col IV from human placenta (MilliporeSigma, Oakville, ON, Canada) to facilitate adherence. Cells were maintained in physioxia at 37 °C, 5% CO_2_, 5% O_2_, 90% N_2_, and 80% relative humidity in a Heracell VIOS 160i tri-gas incubator (Thermo Fisher Scientific, Mississauga, ON, Canada). At 70%–80% confluence, MSCs were washed briefly with Dulbecco’s phosphate-buffered saline without calcium and magnesium (DPBS^(−/−)^) (MilliporeSigma, Oakville, ON, Canada) and enzymatically detached by 2 min–3 min incubation with TrypLE Select (Thermo Fisher Scientific, Mississauga, ON, Canada). Upon complete dissociation, an equal volume of media was added to the cell suspension, and the cells were enumerated using the CellDrop Fluorescent Cell Counter (DeNovix Inc., United States). Cells were pelleted by centrifugation at 150 *g* for 5 min, and the cell pellet was resuspended in fresh chemically defined media before re-seeding. All experiments were performed using MSCs between mPDLs 16–24.

### 2.3 Self-assembled spheroids

Spheroids were generated using a protocol adapted from the study by Li et al. (2021): 96-well plates were coated with 75 µL of sterile 1% agarose (w/v) and allowed to solidify at room temperature. The coated plates were used immediately or stored up to 1 week in 80 µL DPBS^(−/−)^ per well. DPBS^(−/−)^ was aspirated prior to seeding. MSCs were suspended in NutriStem XF® culture media at a concentration of 437,500 cells/mL (35,000 cells/well). A measure of 80 μL of cell suspension was added to the coated wells, and spheroids were allowed to spontaneously aggregate over 4 days in the incubator. To generate spheroids of different sizes, UC MSCs were cultivated using initial cell counts of 7,000, 15,000, 35,000, or 75,000 MSCs per well.

### 2.4 Morphometric analysis

To quantify the differences in spheroid morphology across MSC tissue sources, AD, BM, PL, and UC MSC spheroids were nucleated using 35,000 cells/well. Phase contrast images were captured on days 1, 2, and 4 using an ECHO Revolve inverted microscope (ECHO Inc., San Diego, CA, United States) with ×10 objective for morphometric analysis. Images were acquired on days 1, 2, and 4 and processed using Adobe Photoshop 2023 (24.0.1, Adobe, San Jose, CA, United States). Spheroids were selected using the Object Selection tool. In cases where the background was too high for auto-selection, the Magnetic Lasso tool was used to manually trace the spheroid’s perimeter. The background was replaced with gray as a separate layer using Photoshop to restrict the calculations of each spheroid. For the initial spheroid seeding density experiments, the Ruler tool was used to measure the spheroid’s diameter after 4 days in culture. Next, the Measure tool was used to automatically quantify the spheroid’s area, perimeter, and circularity. At least 19 spheroids per biological replicate, per tissue source, were quantified.

### 2.5 Multilayered MSC sheets

MSCs were expanded and maintained to harvest an appropriate number of cells for the experiments. High-density MSCs (7,813 cells/cm^2^) were seeded into 24-well plates in chemically defined media with media changes every 2–3 days and incubated at 37 °C, 5% CO_2_, 5% O_2_, 90% N_2_, and 80% relative humidity. At specified time-points (days 1, 2, 4, or 7 after seeding), MSCs were washed with 1X DPBS^(−/−)^ and then decellularized (see Section 2.6) for subsequent immunostaining.

### 2.6 Decellularization

Spheroids and multilayered sheets were washed twice in 1X DPBS^(−/−)^ for 5 min each and then incubated in the decellularization solution (20 mM NH_4_OH + 0.5% Triton X-100 plus 1X cOmplete™ EDTA-free protease inhibitor cocktail) (Roche, Mississauga, ON, Canada) for 30 min at 37 °C, 5% CO_2_, 5% O_2_, and 80% relative humidity. To remove the residual DNA, the decellularized spheroids were washed twice more in 1X DPBS^(−/−)^ and then treated with 50 kU of DNAse I plus EDTA-free protease inhibitor diluted in water for 30 min at 37 °C, 5% CO_2_, 5% O_2_, 90% N_2_, and 80% relative humidity. The washes were repeated, and then the spheroids were stored at 4 °C for whole-mount immunofluorescence or submerged in 75 µL radioimmunoprecipitation (RIPA) buffer and immediately flash-frozen in liquid nitrogen and stored at −80 °C until analyzed by Western blot.

To assess the retention of cellular material after decellularization, untreated MSC sheets were immunostained with anti-carboxymethyl-lysine (anti-CML; 1: 200; ab125145; Abcam, Waltham, MA, United States), as detailed in Section 2.7. The sheets were imaged and then decellularized and re-imaged (Supplementary Figure S1A). All the images were captured using a Nikon ECLIPSE Ti2-E inverted microscope with the ×20 objective lens.

Retention of nuclear material after decellularization was assessed using FIJI analysis of DAPI-stained decellularized matrices (Supplementary Figure S1B). DAPI 16-bit images were imported, and thresholds with a black background were set for the cellularized (913–65535) and decellularized (1600 or 2724–65535) matrices, respectively. A Gaussian blur with a sigma factor of 2 was applied, with auto thresholding set to “default dark.” The images were masked based on the thresholding values, and the “analyze particles” tool was used to obtain the nuclei count. Intact nuclei were counted from at least 10 fields of view for each of the three decellularized wells and one cellularized well in each experiment (Supplementary Figure S1C). Decellularization efficiency was calculated as the percentage of the nuclei in the decellularized wells compared to that in the cellularized wells.

### 2.7 Immunofluorescence

Spheroids and sheets were immunostained in 1.5 mL micro-centrifuge tubes with nutation or in-well, respectively. For confocal imaging, UC MSCs were seeded at 4,000 cells/cm^2^ onto coated IBIDI 8-well chamber slides. Cellularized samples (Supplementary Figure S1) were fixed in 10% neutral buffered formalin (MilliporeSigma, Oakville, ON, Canada) for 10 min, rinsed in 1X DPBS^(−/−)^, and then permeabilized with PBT (1X DPBS^(−/−)^ + 0.1% Triton X-100) for 10 min. The decellularized sheets and spheroids were not fixed prior to immunostaining. Following three washes in 1X DPBS^(−/−)^, the samples were blocked with 10% normal donkey serum (MilliporeSigma, Oakville, ON, Canada) diluted in PBST (DPBS^(−/−)^ + 0.1% Tween-20) for 30 min. Primary antibodies against human collagen IV (Col IV, 1: 200, Bio-Techne, Toronto, ON, Canada, NB120-6586; Col IV alpha 1 NC1, 1: 100 Chondrex, Inc., Redmond, WA, United States, 7070), laminin (LM, 1: 200, Bio-Techne, Toronto, ON, Canada, NB300-144), fibronectin (FN, 1: 200, Bio-Techne, Toronto, ON, Canada, AF1918), collagen VI alpha 1 (Col VI, 1: 200, Bio-Techne, Toronto, ON, Canada, NB120-6588), or lysyl oxidase-like 2 (LOXL2, 1: 100, Abnova, Taiwan, 675-773) were diluted in the antibody dilution buffer (PBS + 0.1% Triton X-100 with 1% bovine serum albumin (BSA)) (MilliporeSigma, Oakville, ON, Canada) and incubated with the sample overnight at 4 °C. Scaffolds were washed thrice for 5 min each with 1X DPBS^(−/−)^ at room temperature and then incubated for 1 h at room temperature with secondary antibodies [donkey anti-rabbit IgG (H + L) conjugated to Alexa Fluor™ 647 (1: 1,000, Invitrogen, Burlington, ON, Canada, A32795), goat anti-rat 1gG (H + L) conjugated to Dylight™ 680 (1: 500, Bio-Techne, Toronto, ON, Canada, NBP2-68483FR), or donkey anti-Sheep IgG conjugated to Alexa Fluor™ 594 (1: 1,000, Invitrogen, Burlington, ON, Canada, A110106)] in the antibody dilution buffer. The samples were rinsed twice with 1X DPBS^(−/−)^ and then stained with 4′,6-diamidino-2-phenylindole (DAPI, 1: 4,000, MilliporeSigma, Oakville, ON, Canada) for 2 min at room temperature, followed by three washes for 5 min each in 1X DPBS. Spheroid samples were stored in Invitrogen CytoVista 3D tissue clearing reagent (Invitrogen, Burlington, ON, Canada) until mounting. Animal-derived antibodies and blocking solutions were used in these experiments; we are working on utilizing animal-free replacements such as human serum albumin in future. In addition, we are actively seeking effective biodegradable options to replace Triton X-100, which is extremely toxic to aquatic life, for protocols that require nonionic detergents.

Whole-mount imaging of spheroids was performed using platform-type slides to accommodate the sample depth and prevent deformation under coverslip pressure. No. 2 coverslips were adhered to either end of the microscope slide using clear nail polish to create elevated platforms. The immunostained samples, suspended in CytoVista solution, were pipetted onto the slide and overlaid with a 22 mm × 40 mm No. 1 coverslip. Mounted slides were sealed with clear nail polish and allowed to set overnight before imaging. Confocal images were captured using a Nikon A1R confocal microscope equipped with PlanFluor 10× DIC L N1 and 40× Oil DIC H N2 lenses. Images were analyzed using NIS Elements software (Nikon Instruments, Inc., Melville, NY, United States).

Epifluorescent images of matrix deposition in adherent sheets were captured using a Nikon ECLIPSE Ti2-E inverted microscope with the ×20 objective lens with ×32 averaging on NIS Elements software (Nikon Instruments, Inc., Melville, NY, United States). Images were edited using FIJI (v1.54k; (Schindelin et al., 2012)). Images from wells corresponding to days 1 and 2 were edited to remove the background and visualize weak signals. Raw images were imported into FIJI as 16-bit grayscale images, and the brightness and contrast of each image were optimized; the FN minimum and maximum levels were set at 185 and 400, respectively; the Col IV minimum and maximum levels were set at 170 and 600, respectively; the Col VI minimum and maximum levels were set at 120 and 275, respectively; the LM minimum and maximum levels were set at 115 and 540, respectively. Images taken on days 4 and 7 were comparably adjusted to maintain consistency between the time-points. The FN minimum and maximum levels were set at 160 and 250, respectively; the Col IV minimum and maximum levels were set at 145 and 275, respectively; the Col VI minimum and maximum levels were set at 125 and 200, respectively; the LM minimum and maximum levels were set at 115 and 190, respectively.

### 2.8 Western blot

Total protein was collected from at least 15 pooled, decellularized, and DNAse I-treated spheroids per tissue source and replicate. 3D matrices were solubilized by three rounds of sonication at 10% amplitude for 30 s (1 s on and 1 s off). Protein concentration was determined using bicinchoninic acid assay (BCA; MilliporeSigma, Oakville, ON, Canada) for protein normalization. Samples were diluted and prepared in 6× Laemmli buffer to load 3 µg of total protein in 10 µL per lane and then boiled at 95 °C before loading on an 8% gel (or 12% for elastin). Conventional loading controls such as GAPDH, β-actin, and β-tubulin are intracellular proteins; as they are not present in the decellularized matrix samples, the samples were normalized by loading equivalent total protein (3 µg) per lane. Proteins were subjected to electrophoresis for 1.5 h at 140 V. Size-fractionated proteins were transferred to a nitrocellulose membrane overnight at 4 °C at 30 V. Membranes were washed thrice in 1X Tris-Buffered Saline (TBS) for 10 min each and then blocked for 1 h at room temperature in 5% skim milk or 5% BSA for elastin. The membranes were then incubated with primary antibody solution [rabbit anti-human col IV (1: 1,000, Bio-Techne, Toronto, ON, Canada, NB120-6586), rabbit anti-laminin (1: 1,000, Bio-Techne, Toronto, ON, Canada, NB300-144), rabbit anti-col VI alpha 1 (1: 2,000, Bio-Techne, Toronto, ON, Canada, NB120-6588), sheep anti-fibronectin (1: 3,000, Bio-Techne, Toronto, ON, Canada, AF 1918), or rabbit anti-elastin (1: 3,000, Invitrogen, Burlington, ON, Canada, PA5-99418)] overnight at 4 °C.

Membranes were then washed thrice in 1X TBST for 10 min each. Secondary antibodies [goat anti-rabbit IgG, H + L chain-specific peroxidase conjugate (1: 3,000, MilliporeSigma, Oakville, ON, Canada, 401393), rabbit anti-sheep IgG (H + L) secondary antibody (1: 4,000, Invitrogen, Burlington, ON, Canada, 61-8620), or AMDEX™ goat anti-rabbit horseradish peroxidase conjugate (1: 3,000, Cytiva, Vancouver, BC, Canada, RPN4301)] were diluted in the blocking solution and added to the membrane for 1 h at room temperature. Membranes were washed four times in 1X TBST and visualized using UltraScence Pico Ultra Western Substrate (FroggaBio Inc., Concord, ON, Canada). Western blots were imaged using an Amersham™ AI600 Imager (GE HealthCare Technologies, United States). Animal-derived antibodies and blocking solutions were used in this study, but we are working toward identifying animal-free replacements such as human serum albumin for future studies.

### 2.9 Recellularization of the matrix sheets

Recellularization of the decellularized matrix sheets was assessed using a sample of pooled UC MSCs from three donors. MSCs cultured in T25 tissue culture flasks were prestained with Calcein Green AM (1: 1,000; Thermo Fisher Scientific, Mississauga, ON, Canada) for 10 min, rinsed with 1× Hanks’ Balanced Salt Solution with Ca^2+^ and Mg^2+^, dissociated using 1× TrypLE, and centrifuged as described in Section 2.2. Calcein Green-labeled MSCs were resuspended at a concentration of approximately 3,900 cells/mL in the culture media. Unfixed MSC-derived sheet matrices were pre-labeled using anti-FN antibody, as described in Section 2.7. The cell suspension was added at a volume of 500 μL/well for 24-well plates and 250 μL/well for 48-well plates and incubated overnight as described in Section 2.2. The next day, the culture vessels were placed in a temperature- and humidity-controlled stage-top incubator (TOKAI HIT, Co., Ltd., Japan). Time-lapse images of cell–matrix interactions were captured every 5 s over a 2-min period using the Nikon ECLIPSE Ti2-E inverted microscope (Nikon Instruments, Inc., Melville, NY, United States) under a ×20 objective.

### 2.10 Statistical analyses

Statistical analysis was carried out using Prism 10.3.1 (GraphPad Software, LLC, San Diego, CA, United States). Significant differences were calculated using Student’s t-test (decellularization efficiency), two-way analysis of variance (spheroid morphometrics), and Kruskal–Wallis test (spheroid initial seeding density). Error bars represent the standard deviation (SD) ± of the calculated means. Here, ns = nonsignificant, P > 0.05, * P ≤ 0.05, ** P ≤ 0.01, *** P ≤ 0.001, and **** P ≤ 0.0001. All the experiments were performed three independent times, except for immunostaining of whole-mount spheroid scaffolds, which was performed using 2–3 technical replicates.

## 3 Results

### 3.1 MSCs from different human tissues self-assemble into 3D spheroids in chemically defined media

**FIGURE 1:**
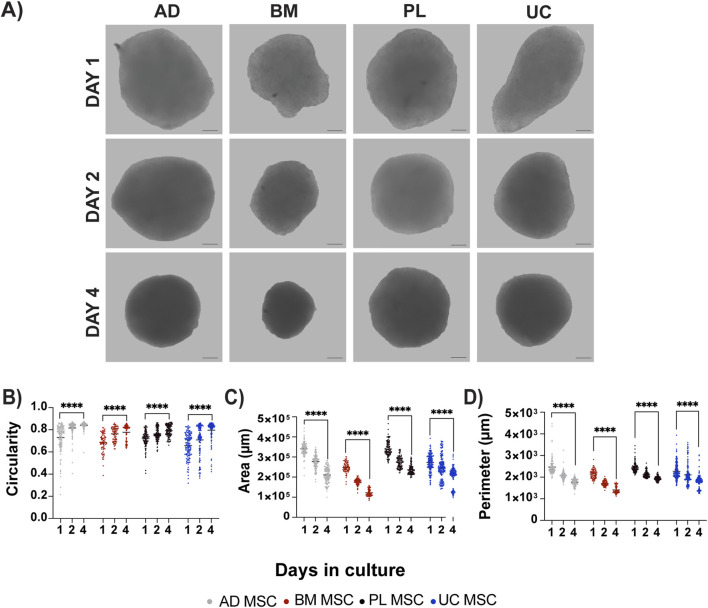
Scaffold-free MSC spheroids exhibit features of self-directed organization. Morphometric analysis of self-assembled MSC spheroids in defined media shows **(A)** a visible increase in uniformity that can be quantified by **(B)** increased circularity (1.0 is a perfect circle), **(C)** reduced area, and **(D)** decreased perimeter, which together are the hallmarks of tissue compaction. Adipose (AD), bone marrow (BM), placental (PL), and umbilical cord (UC) MSCs (scale bar = 100 µm; **** p < 0.0001).

We first analyzed whether MSCs from adult (AD and BM) and perinatal (PL and UC) tissues could reliably form spheroids with an organized matrix in animal-component-free and chemically defined conditions. Within 4 days of high-density seeding on agarose-coated 96-well plates, MSCs from both adult and perinatal tissues formed a single free-floating spheroid in each well (Figure 1A). A small number of unincorporated cells remained in the solution and formed micro-aggregates or attached to the wall of the well, particularly in AD MSC cultures (not shown).

Spheroid geometry influences ECM synthesis and organization both directly (Gonzalez-Fernandez et al., 2022) and indirectly through signaling pathways associated with nutrient and oxygen transport (Shearier et al., 2016; Murphy et al., 2017; Kumar et al., 2018), cell–cell interactions (Im et al., 2021), and cytoskeletal tension (Zhou et al., 2017). Accordingly, we quantified spheroid morphometrics (diameter, area, and circularity) over time to assess whether changes in the matrix content are associated with, or potentially driven by, alterations in the spheroid structure.

Over time, the spheroids became increasingly uniform in size and morphology. Circularity (where 1.0 is a perfect circle) increased from day 1 to day 4 by 11%–15% for each MSC type, leading to average circularities of 0.84, 0.78, 0.80, and 0.80 by day 4 for AD, BM, PL, and UC MSCs, respectively (Figure 1B). Circularity was accompanied by compaction, which was evidenced by a significant decrease in the area (Figure 1C) and perimeter (Figure 1D). Spheroids that were much smaller or larger than the mean on day 1, which may not have aggregated as efficiently or in which there were cell dispensing errors, remained outliers over the time course although their morphometric changes followed the other spheroids.

**FIGURE 2:**
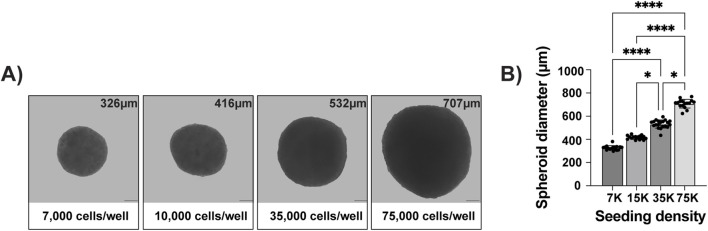
Spheroid diameter correlates with seeding density. Spheroids were seeded in defined media using 7,000, 15,000, 35,000, or 75,000 UC MSCs and measured after 4 days. **(A,B)** Spheroid size correlated with seeding density, but it was not in a linear fashion. Average spheroid diameter (μm) (A, upper right corner) was calculated from **(B)** 15 spheroids per group. Spheroid size was highly consistent between replicates. Representative images are from n = 3 independent experiments (scale bar = 100 μm; * p < 0.5 and **** p < 0.0001).

Thus, we next analyzed how the initial cell density might influence the spheroid self-assembly. Spheroids were nucleated using 7,000, 15,000, 35,000, and 75,000 MSCs per well (Figure 2A). Irrespective of the seeding density, all the spheroids successfully self-aggregated. The spheroid size increased relative to the seeding density, but it was not linear (Figure 2B). All spheroids were visible to the naked eye, with diameters ranging from 326 to 707 µm (Figure 2A). Excluding the largest aggregates nucleated with 75,000 MSCs, the spheroids could be aspirated using a P1000 pipette without shearing. On the other hand, the smallest spheroids were challenging to visualize and manipulate. Considering these practical limitations and the reported oxygen diffusion limits (Murphy et al., 2017), we selected spheroids nucleated using 35,000 MSCs and cultured to day 4 for the remainder of the study.

### 3.2 MSC spheroids produce Col IV-positive matrices in chemically defined media

We then analyzed whether the MSC spheroids cultivated in the defined media contained basement membrane-type Col IV networks. Spheroids provide a physiologically relevant culture context in which 3D cell–cell interactions and biomechanical cues more closely resemble the tissue environment, in contrast to rigid two-dimensional (2D) substrates that can bias MSCs toward fibrotic matrix production (Walker et al., 2021; Loomis et al., 2022; Park et al., 2023). Moreover, the spheroid format enables the assessment of whether MSC-secreted ECM proteins are appropriately processed and assembled into organized, tissue-like basement membrane scaffolds.

**FIGURE 3:**
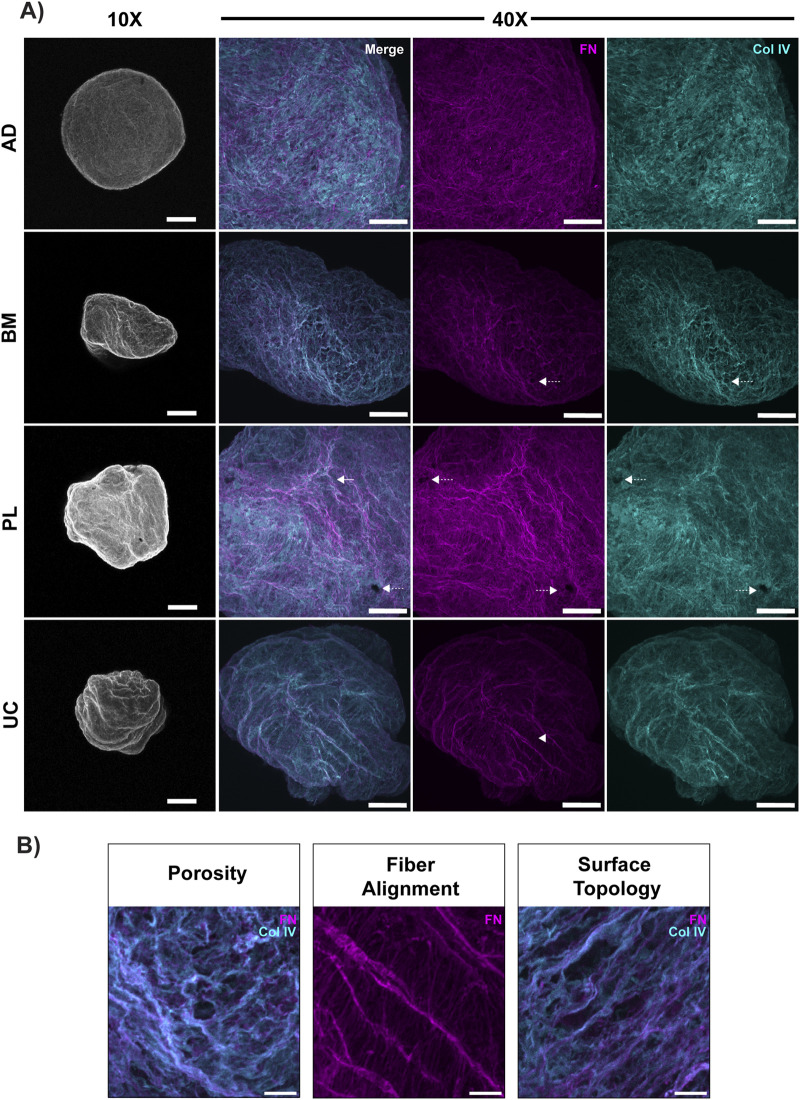
MSC spheroids produce Col IV-positive matrices in chemically defined media. Whole-mount confocal images of 4-day-old decellularized matrices produced by spheroid cultures of MSCs from adult (adipose, AD; bone marrow, BM) and perinatal (placental, PL; umbilical cord, UC) tissues. Matrices were immunostained for fibronectin (FN, magenta) and Col IV (cyan). **(A)** Maximum intensity projection of 2-μm confocal images captured over a Z-depth of 50 μm with a ×10 objective. Overlaid FN and Col IV confocal images, shown in greyscale, show the surface roughness. Some loss of circularity and folding may be due to compression between the slide and the coverslip, indicating that the scaffolds have plasticity. Scale bar = 50 μm. The maximum intensity projection of 0.5-μm slices captured over a Z-depth of 80 μm with a ×40 objective show complex networks of FN and Col IV. These proteins appear to be aligned in a complementary fashion that moderately overlaps (white). Architectural features including pores (dotted arrow), fiber directionality (arrowhead), and surface topology (arrow) are also apparent. **(B)** Digital magnification of the representative panels in **(A)** show enhanced architectural details, including defined pore structures (porosity), alignment of FN fibers across two planes (fiber alignment), and rough surface topography featuring peaks and troughs. These topological and topographical features are found in all the examined spheroid-derived matrices. Representative images from 3 to 4 technical replicates per MSC tissue source (scale bar = 100 μm).

Spheroids were decellularized using a protocol that was reported to preserve matrix architecture (Li et al., 2021) and immunostained for FN and Col IV. Multi-labeling for additional basement membrane constituents was impeded by the lack of reliable commercial antibodies produced in other species. Confocal imaging of the spheroids at low magnification (×10) confirmed the tissue-dependent size differences documented by light microscopy (Figure 1) but revealed complex surface topology, some of which may be caused by deformation after mounting, which implies viscoelasticity (Figure 3A). At higher magnification (×40), complementary networks of FN and Col IV were observed (Figure 3B). All the decellularized matrices presented architectural basement membrane features, including pores, fiber alignment, and rough surface topography (Figure 3A), which are more clearly depicted in the digitally enlarged representative images (Figure 3B).

**FIGURE 4:**
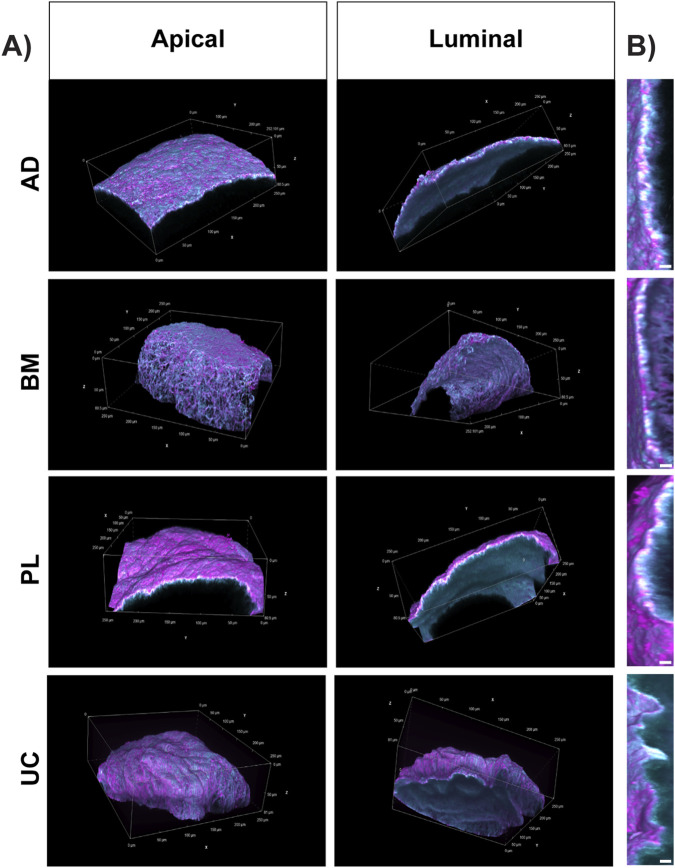
Perinatal-derived MSCs produce self-assembled matrices with overlaid FN and Col IV sheets. Decellularized whole-mount spheroid-derived matrices produced by adipose (AD), bone marrow (BM), placental (PL), and umbilical cord (UC) MSCs were examined in the volume view with alpha blending to highlight the surface and compositional topography. **(A)** Fibronectin (FN, magenta) and Col IV (cyan) appear to be interwoven in matrices produced by adult MSCs (AD and BM). In contrast, the perinatal MSCs (PL and UC) establish matrices with sheet-like organization, whereby FN is enriched on the apical surface, complemented by a sheet of Col IV on the underlying luminal surface. **(B)** Cross-sections reveal a marked overlap of FN and Col IV (white) at the interface of the apical and luminal sheets (scale bar = 5 μm). Representative images from 3 to 4 technical replicates per MSC source. Confocal images were captured in 0.5-μm sections through a Z-depth of 80 μm.

3D volumetric reconstruction of the confocal images showed distinct patterns of matrix organization. In the adult MSC-derived matrices, FN and Col IV appeared to be interwoven, whereas the perinatal MSC-derived matrices segregated FN into apical sheets, with an underlying sheet of Col IV (Figure 4A). Regardless of their relative orientation, fibronectin and Col IV rarely overlapped, except at the interface between the apical and luminal matrix facets (Figure 4B).

### 3.3 Self-assembled MSC matrices contain canonical basement membrane factors

**FIGURE 5:**
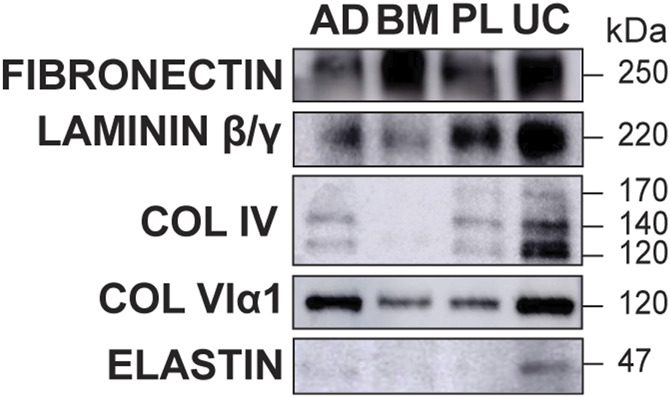
MSC-derived matrices contain basement membrane-specific proteins. Relative abundance of basement membrane proteins in decellularized adipose (AD), bone marrow (BM), placental (PL), or umbilical cord (UC) MSC spheroids were analyzed by Western blot. Samples were normalized by total protein (3 μg/lane). UC MSC-derived matrices were enriched for the basement membrane factors Col IV, Col VIα1, and elastin. Matrix from BM MSCs contained relatively more fibronectin (FN) than the other scaffolds. Representative data from n = 3 independent experiments.

Having shown that MSC-derived matrices contain networked FN and Col IV, we used Western blot analysis to assess whether other basement membrane factors were also present. Decellularized spheroids were solubilized, normalized to total protein, size-fractionated, and then immunoblotted for FN, LMβ/γ, Col IV, Col VIα1, and elastin (Figure 5). LM β and γ chains, along with an α chain, form the basic heterotrimeric structure of all LMs. They assemble with other proteins, such as Col IV and nidogen, to create the complex, organized network of the basement membrane (Lennon and Sherwood, 2025; Page-McCaw and Ferrell, 2025; Page-McCaw et al., 2025). Col VIα1 is a key component of type-VI collagen, which acts as a structural link between the basement membrane and the surrounding interstitial matrix, providing mechanical support to tissues (Cescon et al., 2015; Gregory et al., 2024). Elastin anchors cells to the matrix and provides mechanical support; it functions in conjunction with other basement membrane components to provide elasticity, allowing tissues to stretch and recoil (Trębacz and Barzycka, 2023; Ruiz et al., 2024; Krymchenko et al., 2025). The relative abundance of these analytes differed in matrices produced by each MSC donor. BM MSC-derived matrices were markedly enriched with FN, whereas UC MSC-derived matrices were enriched with all the basement membrane constituents compared with those of other donors (Figure 5). Col IV and elastin were less abundant in the AD-derived scaffolds, whereas all the analytes except elastin were detected in PL-derived matrices (Figure 5).

### 3.4 Multilayered UC MSCs produce basement membrane-type sheets in defined media

Having determined that the UC MSC population synthesizes the most robust repertoire of basement membrane proteins when cultured as spheroids (Figure 5), we next analyzed whether a similar basement membrane-type matrix could be generated when the UC MSCs were grown as adherent cultures. Compared with spheroids, multilayered cell sheets are technically simpler and may offer greater reproducibility and scalability. However, adherence to rigid culture substrates imposes a stiffer microenvironment and reduces 3D cell–cell interactions, which are conditions that are known to influence matrix deposition and may preferentially promote interstitial rather than basement membrane matrix production. We, therefore, sought to determine whether basement membrane assembly is preserved under these 2D culture conditions.

**FIGURE 6:**
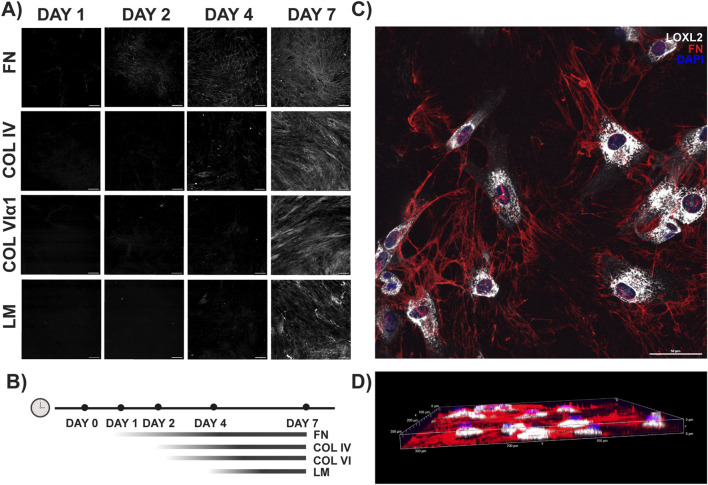
Multilayered UC MSCs construct sheets of scaffolded matrix. **(A)** Fibronectin (FN) is first detected by immunogenic staining 2 days after seeding, followed by Col IV on day 4. Col VIα1 and laminin (LM) are not readily detected until day 7, when all four analytes are visible components of a networked matrix structure (scale bar = 100 μm). **(B)** Schematic summarizing the temporal incorporation of extracellular matrix components depicted in **(A)**. **(C)** Maximum intensity projection of 4-day MSC multilayers show networks of fibronectin (FN; red) intercalating between MSCs expressing high levels of the Col IV cross-linking enzyme LOXL2 (white) in **(D)** a 8-μm-thick matrix. Data shown are representative from a dataset of n = 3 independent experiments, with 5–6 fields of view per experiment.

UC MSCs were cultured in adherent multilayers followed by decellularization to assess ECM biogenesis. We then analyzed the deposition of select adhesive (FN and LMβ/γ) and structural (Col IV and Col VIα1) basement membrane proteins over time by immunofluorescence. FN was the most prevalent matrix component on day 1 and increased in abundance and architectural complexity through day 7 (Figures 6A,B). Col IV and Col VIα1 were barely detectable on day 2 and continued to accumulate in networked structures through day 7 (Figures 6A,B). On day 7, LMβ/γ was the last analyte to be detected. The matrix sheets were 8 µm–10 µm thick on day 7, as determined using cross-sections of Z-stacked images (Figure 6C). In cellularized samples, networked FN (red) is seen connecting with and intercalating between MSCs (white) highly expressing the Col IV cross-linking enzyme lysyl oxidase-like 2 (LOXL2; Figures 6C,D).

### 3.5 UC MSCs matrix sheets are amenable to recellularization

Finally, we analyzed whether MSC-derived matrices were amenable to recellularization and could support cell–matrix interactions. Recellularization on spheroid matrices was inefficient, as many of the suspended cells promptly adhered to the walls of the well plate. Matrix sheets were more practical and efficient than spheroids for reseeding and were selected for this experiment. We evaluated the cell–matrix interactions using decellularized sheets that were 8 µm–10 μm thick. This assay format also allowed us to capture time-lapse images of cell migration through the Z-plane of the matrix, which would not have been possible with suspended spheroids.

**FIGURE 7:**
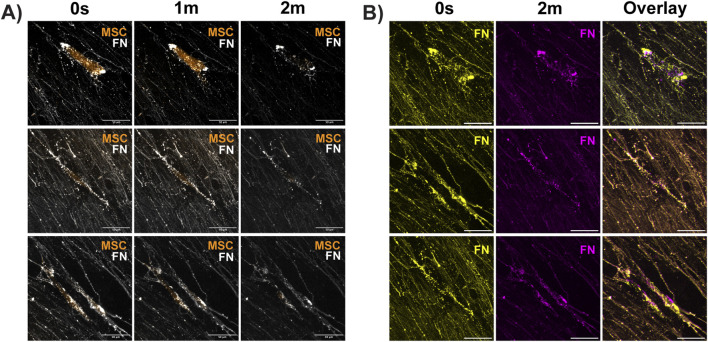
UC MSC-derived matrix sheets can be colonized by new cells. Decellularized umbilical cord (UC) MSC sheets produced in chemically defined media support interactive cell migration of newly introduced UC MSCs (Supplementary Movie S1). **(A)** In representative time-lapse images, MSCs (orange) can be seen moving through the Z-plane of scaffolded fibronectin (FN; white). **(B)** FN scaffolds were false colored at two time-points (0 s, yellow; 2 min, magenta) and overlaid to detect FN movement. The presence of unblended FN signal in cell-occupied regions shows the relocation of FN, indicating dynamic cell–matrix interactions. Blended color (orange) in the unoccupied scaffold areas indicates that FN remains static in the absence of cell movement (scale bar = 50 µm).

Fluorescent-labeled UC MSCs were incubated overnight with decellularized matrix sheets that were immunostained to visualize FN. The next day, cell–matrix interactions were assessed by time-lapse imaging. MSCs successfully adhered to the nascent scaffolds (Figure 7). Time-lapse microscopy showed MSCs attached to the FN fibers. MSCs appeared to leverage this matrix interaction to migrate through the focal planes of the matrix (Figure 7A) by pulling or tugging on the flexible FN fibers (Figure 7B, Supplementary Movie S1). In preliminary studies, the sheet matrix and other matrices produced by MSCs in our animal component-free workflow can support the adhesion, migration, and proliferation of other cell types, including human neural progenitor cells, human umbilical vein endothelial cells, and fibroblasts (not shown). Taken together, in this study, we establish MSCs as a viable and ethically favorable source for the generation of self-assembling matrix proteins, including basement membrane components, in chemically defined conditions.

## 4 Discussion

There is a critical need for functional human matrix components to support the next generation of high-fidelity tissue models for experimental use and transplant (Catarino et al., 2022; Liu et al., 2023; Naderi-Meshkin et al., 2023; Schneider et al., 2023). Moreover, there is a paucity of intact and functional basement membrane biomaterials to recapitulate the complexity of human tissues, and to facilitate vascularization (Chan et al., 2016; Marrella et al., 2018; Minor and Coulombe, 2020; Zhao et al., 2022). Here, we report that human MSCs, which occupy perivascular niches *in vivo* (Kang et al., 2010; Corselli et al., 2013; Caplan, 2017), can be utilized to synthesize functional basement membrane components in a defined, animal component-free bioprocess. The development of an animal component-free platform based on human MSCs offers a renewable and physiologically relevant strategy for scalable ECM production, reducing the reliance on ethically contentious animal-derived materials, lowering the environmental burden associated with animal agriculture, and minimizing the risk of xenogeneic contamination.

Our findings align with previous studies that demonstrate origin-specific synthesis of ECM factors by MSCs, although this study is not sufficiently powered to statistically correlate the MSC tissue source with ECM composition. In previous transcriptome studies that span multiple MSC donors and origins, we found that UC MSCs express significantly higher levels of the non-fibrillar basement membrane collagens (Col IV and Col VI) than fibroblasts (Wiese and Braid, 2020) and BM MSCs (Wiese et al., 2022). Similarly, Ragelle et al. (2017) found that decellularized neonatal dermal fibroblast-derived matrices possessed less total collagen than AD and BM MSC-derived scaffolds. The most abundant structural components in those BM MSC-derived scaffolds were Col I and Col VI, whereas Col II, III, and IV were present at lower levels (Ragelle et al., 2017). These results were consistent across all five donors (Ragelle et al., 2017). Another group reported that decellularized BM MSC scaffolds contained Col IV, whereas AD MSC scaffolds did not (Marinkovic et al., 2020), which contradicts our findings. Amable et al*.* compared the ECM components secreted by AD, BM, and UC MSCs and found that UC MSCs did not produce detectable amounts of LM, FN, Col II, or Col IV. In their study, AD MSCs were the only MSC population to secrete Col IV at detectable concentrations, and they also produced more Col I, II, and III than BM and UC MSCs (Amable et al., 2014). It is plausible to expect that these disparate outcomes are because of the different media formulations used in these studies, including FBS-supplemented (Bartosh et al., 2010; Amable et al., 2014; Cesarz and Tamama, 2016; Lee et al., 2016; Ragelle et al., 2017; Marinkovic et al., 2020; Chiang et al., 2021; Fuentes et al., 2022; Yin et al., 2024; Chen et al., 2025), serum-free (Rakian et al., 2015; Choi et al., 2021), and xeno-free products (Bauman et al., 2018; Parvin Nejad et al., 2023). In this study, we aimed to test whether MSCs are capable of synthesizing basement membrane-type matrices under chemically defined culture conditions that exclude the rich array of growth factors, adhesion factors, and stabilizing components found in serum- or hPL-supplemented media (Hodges and Melcher, 1976; Oikonomopoulos et al., 2015; Karnieli et al., 2017; Klinker et al., 2017; Delabie et al., 2025).

In mammals, Col IV is encoded by a family of six genes (*COL4A*1–6) that produce discrete Col IV α-chains (α1–α6) (Page-McCaw and Ferrell, 2025; Page-McCaw et al., 2025). These α-chains can produce three supramolecular scaffolds, namely, Col IV^α121^, Col IV^α345^, and Col IV^α121–α556^, which form networks with different structural and biomechanical properties (Page-McCaw and Ferrell, 2025; Page-McCaw et al., 2025). The expression of the *COL4A* genes differs between tissues and shifts during development, causing the formation of functionally specialized basement membranes throughout the body (Page-McCaw and Ferrell, 2025; Page-McCaw et al., 2025). The observation that MSCs from different tissues produce distinct Col IV α-chains was, therefore, not unexpected and is consistent with published reports. For example, we previously compared the transcriptome profiles of three donors each of UC and BM MSCs and their responses to cytokine stimulation. In that study, unstimulated BM MSCs, as used here, expressed very high levels of *COL4*A1 and *COL4*A2 and very low levels of *COL4*A5, and they did not express *COL4*A3, *COL4*A4, or *COL4*A6 (Wiese et al., 2022). UC MSCs expressed even higher levels of *COL4*A1 and *COL4*A2, moderate levels of *COL4*A5, and low levels of *COL4*A3, *COL4*A4, and *COL4*A6 (Wiese et al., 2022). Notably, the expression of these genes did not vary significantly between the donor populations and were consistent with an earlier study using other UC MSC donors cultured in a different media formulation (Wiese et al., 2019). These data indicate that Col IV synthesized by BM MSCs may only form Col IV^α121^ scaffolds, whereas UC MSCs synthesize the starting material for all three Col IV supramolecular structures, warranting further study.

These unique properties may contribute to the apparent differences in volumetric Col IV scaffolding, where the Col IV and FN networks constructed by BM MSC spheroids (Figure 3A, second row) appeared to be less dense than the perinatal MSC-derived matrices (Figure 3A, bottom rows). Indeed, the highly stringent glomerular basement membrane in the kidney, which filters urine to eliminate waste while retaining proteins and other useful molecules, contains two Col IV networks, namely, a Col IV^α121^ network and a Col IV^α345^ network (Page-McCaw et al., 2025). Our gene-expression data indicate that UC MSCs can synthesize both networks, along with Col IV^α121–α556^, which would yield a more stringent Col IV network than the BM MSCs, which can only produce a Col IV^α121^ network (Wiese et al., 2022).

Compaction is a natural developmental process that indicates organotypic self-organization (Baker and Chen, 2012; Domnina et al., 2020). Spontaneous compaction, as observed in this study (Figure 1), is driven by ECM structure and stiffness (Efremov et al., 2021; Jahin et al., 2023; Kosheleva et al., 2023), actin contractility (Sart et al., 2014; Tsai et al., 2015; Turlier and Maître, 2015; Lee et al., 2019; Sharma et al., 2025), cell–matrix adhesion (Bartosh et al., 2013; Yokomine et al., 2025), and the availability of matrix remodeling enzymes (van der Net et al., 2024; Yokomine et al., 2025). Here, we found that the BM MSC-derived spheroids were significantly more compact than the perinatal or AD-derived MSC spheroids (Figure 1), which could be caused by several key differences. The protein composition of the BM MSC matrices varied the most from that of the other tissue sources (Figure 5), and they also showed a more open network of FN and Col IV (Figures 3A, 4A). These data indicate that the BM MSC-derived matrices may have different biomechanics than the matrices derived from the AD, UC, and PL MSC donors, which we are currently assessing using biophysical techniques such as atomic force microscopy and optical tweezers.

The rate of cell proliferation may also differ between MSC spheroids, leading to differences in cell density, with consequent effects on compaction. A previous study showed that as the number of MSCs per spheroid increases, the packing density decreases, leading to the formation of tightly packed, smaller spheroids and loosely packed, larger spheroids (Murphy et al., 2017). It is possible that variations in the proliferation rates between spheroids, and thus differences in cell number over time, correlate with their compaction. In addition, analysis using spheroids derived from numerous donors of each sex would help discern whether different compaction rates are caused by tissue-imprinted variables or biological differences between the donors irrespective of the tissue of origin.

We performed a temporal analysis of ECM deposition by MSCs cultured in multilayered sheets at different time-points, studies that are reportedly under-represented in the literature (Fuentes et al., 2022). A previous study using human AD MSCs cultured in DMEM/F12 plus FBS found that matrix assembly was slower in sheet cultures than in spheroids, whereby LM and Col IV were barely detectable in the ECM after 4 days although the MSCs stained brightly, indicating that the secretion of these proteins had just initiated (Glazieva et al., 2023). This is in agreement with our data, where Col IV was readily detected by ICC in matrices isolated from 4-day-old spheroids (Figure 3) when it was barely discernible in matrices from 4-day-old MSC multilayers (Figures 6A,B). Despite the relative delay in matrix assembly, the multilayered MSC sheets established their ECM in a temporal sequence reminicent of developmental basement membrane assembly, starting with an FN network (Figures 6A,B). This foundational scaffold was then elaborated with LM, Col IV, Col VI, and additional FN, recapitulating the hierarchical pattern of basement membrane assembly *in vivo* (Pompili et al., 2021). In-depth characterization of the MSC-derived matrices using proteomics is necessary to fully appreciate and compare the complement of the adhesion, structural, proteoglycan, instructive, and signaling components and their assembly. However, the analysis of decellularized matrices is not straightforward as conventional preparation procedures can lead to artefacts in the data, whereas matrisome analysis from cellularized samples is not sufficiently enriched to detect proteins with low abundance (Byron et al., 2013; Ouni et al., 2020; Monteiro-Lobato et al., 2022; Naba, 2023; Diedrich et al., 2024; Frattini et al., 2024).

Environmental conditions such as oxygen tension also influence matrix synthesis (Lee et al., 2016; Satyam et al., 2016; Kumar et al., 2018). For example, a recent study showed that human articular chondrocytes cultured at physiological oxygen tension (5% O_2_) produced thicker matrices with increased deposition of glycosaminoglycans and Col II, along with increased tensile strength (Dennis et al., 2020). MSC spheroids cultured under hypoxia (2% O_2_) produced more LM, FN, and Col I than spheroids cultured at the ambient (∼20%) O_2_ level (Shearier et al., 2016). As MSCs were cultivated under 5% O_2_ in our study, oxygen tension may be a key differential that contributes to the disparate outcomes between our data and other studies that maintain MSCs at ambient O_2_ tension. MSCs were transiently handled at ambient oxygen levels, which is a potential source of variability in the study; the culture media was also pre-warmed in ambient air. A continuous workflow in an environmentally controlled workstation could potentially improve reproducibility by maintaining the cells in uninterrupted temperature and atmospheric conditions.

## 5 Conclusion

Decellularized matrices derived from MSC spheroids and cell-sheet cultures represent biologically relevant scaffolds that preserve native extracellular matrix architecture, growth-factor-binding capacity, and integrin-specific adhesion cues—features that are essential for supporting cell survival and directing lineage specification in engineered tissues. Here, we provide proof of concept that human MSCs cultured in a chemically defined bioprocess can produce basement membrane components that self-assemble into spheroid and sheet-type matrices. The naturally formed sheet scaffolds can be effective recellularized with other cell types, demonstrating the functional integrity of the MSC-derived matrix proteins. We are currently analyzing whether sub-optimal colonization of the spheroid matrices is because of the differences in the composition or architecture of the 3D and sheet matrices, or simply requires increasing cell–matrix interactions during reseeding. Although purified collagens, including Col I and Col IV, are known to self-assemble into matrices *in vitro* (Kotch and Raines, 2006; Yu et al., 2024; Al-Shaer and Forde, 2025), it is still unclear whether more complex matrices comprising multiple basement membrane proteins can similarly self-assemble outside the cellular context. Collectively, these findings advance the development of high-fidelity, human-derived tissue models for disease modeling, drug discovery, and regenerative medicine, while supporting sustainable and ethically responsible biomedical research.

## Data Availability

The raw data supporting the conclusions of this article will be made available by the authors, without undue reservation.

## Supplementary material

SUPPLEMENTARY FIGURE S1 Ammonium hydroxide decellularization efficiently clears the nuclear and cytoplasmic material. Multilayered UC MSC sheets were decellularized on day 4 **(A)** and day 7 **(C)** after seeding using an ammonium hydroxide protocol. A) Adhered UC MSCs were labeled using DAPI (nucleus) and anti-CML antibody to detect carboxymethyl-lysine residues in the nucleus and cytoplasm. Negligible signal was detected on either channel following ammonium hydroxide-based decellularization, indicating efficient clearance of the nuclear and cytoplasmic material. **(B)** Nuclei were labeled using DAPI and quantified (number in the bottom left of the image). **(C)** The number of nuclei was significantly reduced by 95.8% after decellularization. Representative data from n = 3 independent experiments (scale bar = 100 µm).
