# Supplementary figures and images for: An animal component-free bioprocess for synthesizing 3D human matrix scaffolds using mesenchymal stromal cells
Source: Front Cell Dev Biol. 2026 Apr 13;14:1624745. doi: 10.3389/fcell.2026.1624745 (PMC13111481; doi:10.3389/fcell.2026.1624745)

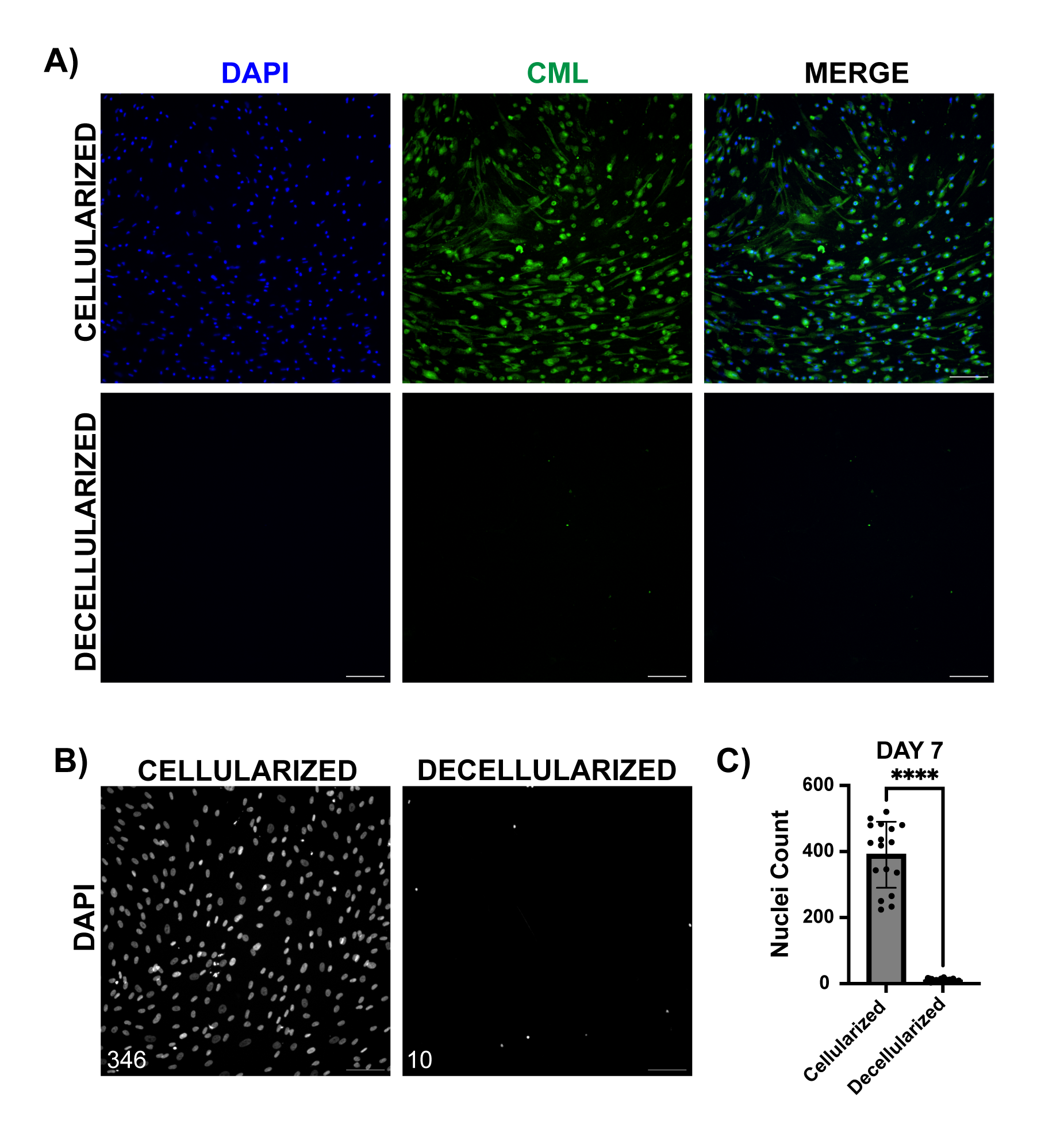

Supplement: Supplementary file 1 [file Image1.tif]
